# Optimal Plant Density Is Key for Maximizing Maize Yield in Calcareous Soil of the South Pannonian Basin

**DOI:** 10.3390/plants13131799

**Published:** 2024-06-29

**Authors:** Ivica Djalovic, P. V. Vara Prasad, Dušan Dunđerski, Snežana Katanski, Dragana Latković, Ljubiša Kolarić

**Affiliations:** 1 Institute of Field and Vegetable Crops, National Institute of the Republic of Serbia, 21000 Novi Sad, Serbia; dusan.dundjerski@ifvcns.ns.ac.rs (D.D.); snezana.katanski@ifvcns.ns.ac.rs (S.K.); 2 Department of Agronomy, Kansas State University, Manhattan, KS 66506, USA; vara@ksu.edu; 3 Faculty of Agriculture, University of Novi Sad, 21000 Novi Sad, Serbia; dragana@polj.uns.ac.rs; 4 Faculty of Agriculture, University of Belgrade, 11000 Belgrade, Serbia; kolaric@agrif.bg.ac.rs

**Keywords:** plant density, maize, yield, South Pannonian Basin

## Abstract

Plant density, the number of plants per unit area, is an important factor in maize production. Plant density exhibits high variability and depends on a number of factors, i.e., the length of the growing period of the hybrid, the morphological characteristics of the plant, the amount and distribution of precipitation during the growing season, the reserve of winter moisture in the soil, the level of soil fertility, the time of sowing, agronomic management practices, and biomass and yield. The objective of this paper was to determine the agronomic optimal plant density for maize in calcareous soil in the semiarid conditions of the South Pannonian Basin. Field experiments were conducted at the experimental field—IFVCNS (two locations: Rimski Šančevi and Srbobran) to evaluate four plant densities (55,000; 65,000; 75,000; and 85,000 plants ha^−1^). The experimental sites “Rimski Šančevi” and “Srbobran” are located in the typical chernozem zone of the southern part of the Pannonian Basin. On average for all hybrids, the grain yield followed a second-degree polynomial model in response to the increasing planting density, with the highest value at plant density (PD2: 65,000 plants ha^−1^). To achieve maximum yield, the optimal planting density for corn hybrids of the FAO 200 group should be 57,600 plants ha^−1^, for the FAO 300 group 64,300 plants ha^−1^, for the FAO 400 group 68,700 plants ha^−1^, for the FAO 500 group 66,800 plants ha^−1^, and for the FAO 600 group 63,500 plants ha^−1^. “Which–Won–Where” biplot showed that the hybrid H24 from FAO 600 group was the highest yielding in all of the environments. Hybrid H17 from the same FAO group was the most stable across all of the environments. Selected hybrids may further be studied for planting density and nutritional requirements for getting maximum yield. By introducing new maize hybrids with higher genetic yield potential and better agronomic management practices, modern mechanization and agricultural techniques allowed to increase planting densities.

## 1. Introduction

Maize (*Zea mays* L.) is one of the most important cultivated plants in the world. It is grown in a relatively wide geographical area and under different climate and soil conditions. In order to make better use of the genetic potential of cultivated varieties or hybrids, they should be grown under the best crop management practices and environments. Today’s maize production is characterized by the use of early hybrids (300 and 400 FAO maturity groups), grown under high sowing densities [1,2]. Plant density, the number of plants per unit area, is an important factor in successful maize production [3]. Timely sowing and the correct sowing density significantly increase crop yields. Plant density exhibits great variability and depends on a number of factors, i.e., the length of the growing season of the hybrid, the morphological characteristics, the growth habit of the plant, the amount and distribution of precipitation during the growing season, the winter moisture reserves in the soil, the level of soil fertility, the sowing time, the crop management, and biomass and yield [4,5,6]. For the above reasons, the optimal density is not a constant value but varies with genotype, environmental conditions, and crop management practices. Hence, differences between potential and realized yields of the genotype or hybrids and their reaction to sowing density should be understood [7]. In maize production, in practice, a significantly lower number of plants per unit area, i.e., per hectare, is often observed in many environments. The causes that lead to a decrease in the number of plants can be various, and the most common are reduced and/or insufficient seed germination and seedling emergence; inadequate pre-sowing soil moisture; sowing outside the optimal time; less favorable agroecological conditions in terms of climatic characteristics and soil properties; the occurrence of diseases and pests during the germination and emergence phases; and early seedling vigor and growth [8]. Any decrease in the number of plants from the optimum leads to a yield reduction because crop density is one of the key prerequisites to obtain high and stable yields.

The Pannonian Basin is a large lowland area in the southeastern region of Central Europe and covers parts of many countries (Austria, Bosnia and Herzegovina, Czech Republic, Croatia, Hungary, Romania, Serbia, Slovakia, Slovenia, and Ukraine). Republic of Serbia is situated in the Central-European, Balkan, Pannonian, and Danube regions. It is the largest agricultural region with a significant portion of crop land, which is primarily rainfed and highly vulnerable to climate with frequent droughts and high temperatures. Serbia is located in the southern part of the Pannonian Basin, and cropland occupies two-thirds of the land area. About 90% of Serbia is in the Danube Basin, and key crops produced in the region include cereals. Maize is the most important cereal, occupying about one-third of crop land. In 2022, Serbia had a maize harvested area of 952,216 ha, with a total production of 4.28 million tons and productivity of 4.49 t/ha (FAO, 2023) [9]. Other important crops of Serbia include wheat, sunflower, soybean, and sugar beet. Maize production in the Pannonian Basin has been negatively affected by extreme weather events (e.g., droughts, dry winds, heat, and cold waves). The increasing frequency of severe droughts over recent decades has led to substantial maize yield losses in the Pannonian Basin in southeastern Europe.

The objective of this paper was to determine the agronomic optimal plant density for maize in calcareous soil in the semiarid conditions of the South Pannonian Basin.

## 2. Results

### Grain Yield and Grain Moisture

The fixed effect tests from the linear mixed effects model (LMM) analysis on grain yield (GY) and moisture are summarized in Table 1. The results indicate significant influences of hybrid (H) and planting density (PD) on the grain yield (*p* < 0.05) and hybrid on moisture (Table 1a). The interactions between hybrid and plant density were not significant for either yield or moisture.

The estimated effects of planting density on grain yield, along with standard errors and 95% confidence intervals, are presented in Table 2. The highest grain yield obtained at PD2 (9.65 t ha^−1^) was statistically similar to yield at PD1 (9.50 t ha^−1^) or PD3 (9.55 t ha^−1^), but significantly higher than yield at PD4 (9.23 t ha^−1^). Overall, the response was quadratic (Table 2).

**Figure 1:**
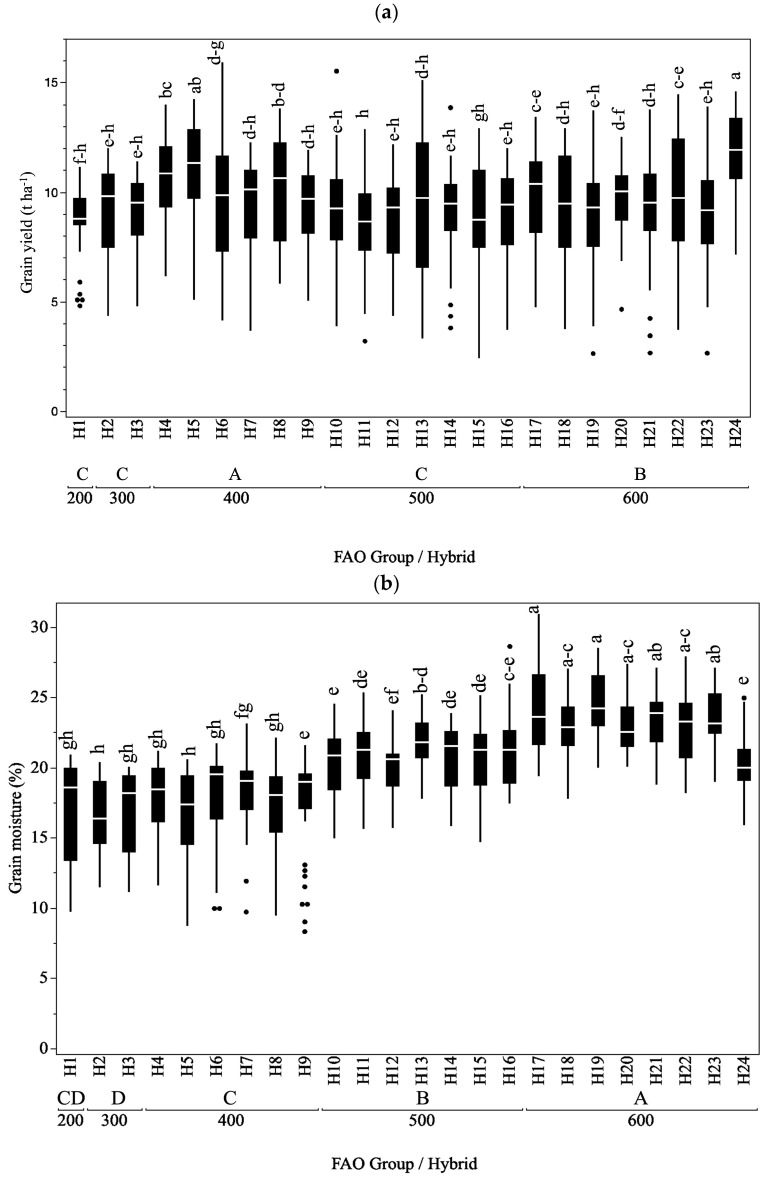
Variability in grain yield (**a**) and grain moisture (**b**) in the hybrids included in the study. Levels not connected by the same letter are significantly different, where smaller letters are attributed to hybrids, while capital letters are attributed to FAO groups.

The 95% confidence intervals for the mean grain yield values ranged from 8.31 to 10.68 (PD1), 8.46 to 10.83 (PD2), 8.37 to 10.74 (PD3), and 8.05 to 10.42 (PD4). The consistent width of the confidence intervals across different planting density levels suggests that the variability in mean grain yield values was consistent across different planting density levels. Grain yield (Figure 1a) and grain moisture (Figure 1b) of hybrids ranged from 2.42 t ha^−1^ for H15 to 15.94 t ha^−1^ for H6, with an average of 9.48 t ha^−1^ and a median of 9.73 t ha^−1^. For grain moisture, the range was from 8.30% for H9 to 30.90% for H20, with an average of 20.26% and a median of 20.40%.

**Figure 2:**
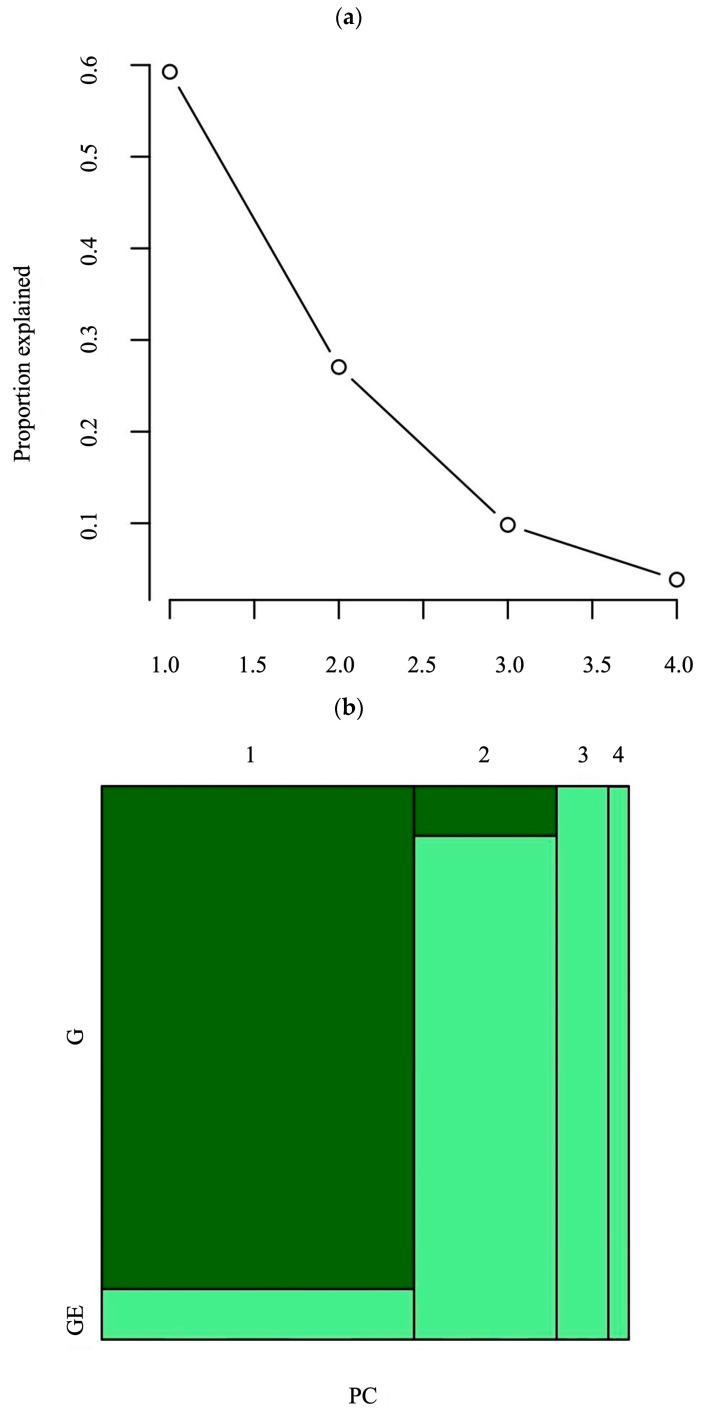

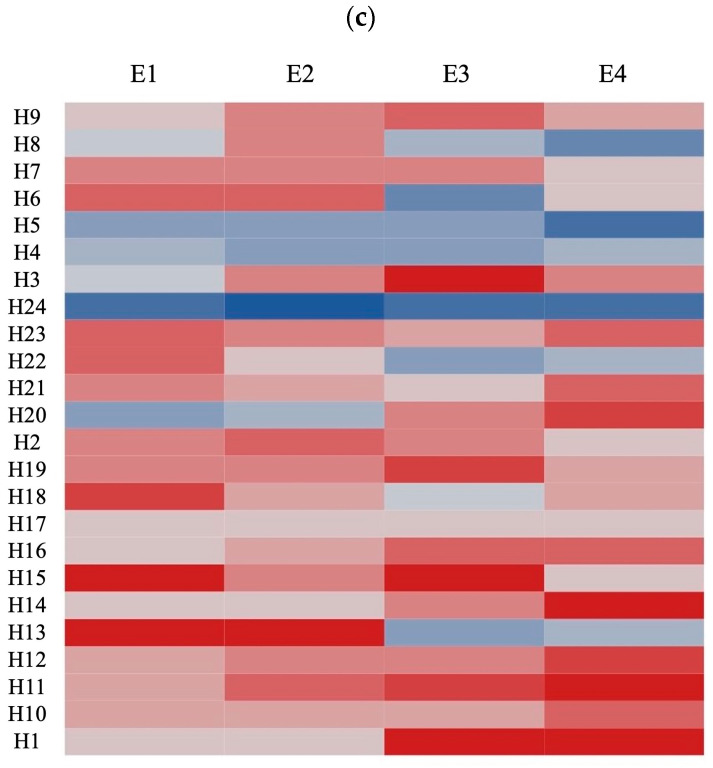
Pre-analysis of GGE for grain yield of 24 maize hybrids in four environments. (**a**) Scree plot with eigenvalues; (**b**) mosaic plot of principal components; (**c**) heatmap plot of GEI. GE—Genotype × Environment; G—Genotype; PC—Principal Component; H1–H24—Genotypes; E1–E4—Environments.

The hybrids were categorized into FAO groups based on the information from seed company catalogs. Within each FAO group, the hybrids were significantly similar to each other in terms of yield (Figure 1a). The first exceptions were seen in FAO 400, where H4 and H5 yielded significantly more than other hybrids, except H8. Based on the width of the box, the H8 had greater yield variability than H4 and H5. The second exception was in FAO 600, where hybrid H24 had significantly higher yield than the rest of the hybrids. On average, the highest grain yield was achieved with hybrids that belonged to FAO 400, significantly higher than other FAO groups (Figure 1a and Figure 2). The comparison between FAO groups 200 and 300 showed a slight increase in yield for group 300, although this difference was not statistically significant (difference = −0.37 t ha^−1^). In contrast, significant differences in yield were observed between groups 200 and 400, with group 400 exhibiting a significantly higher yield compared to group 200 (difference = −1.28 t ha^−1^). Similarly, group 600 showed a significant increase in yield compared to group 200. Group 400 exhibited a higher yield compared to group 500 (difference = 1.04 t ha^−1^, *p* < 0.0001), while group 600 showed a lower yield compared to group 400 (difference = 0.32 t ha^−1^, *p* = 0.0450). Conversely, group 600 exhibited a significantly higher yield compared to group 500 (difference = −0.72 t ha^−1^).

Figure 1b reveals substantial differences between grain moisture of individual hybrids. The lowest grain moisture was seen in H24, and it was significantly different than other hybrids in the FAO 600. There were no significant differences between other hybrids in this group. In the FAO 500, hybrid H12 outperformed other hybrids in this particular group in terms of grain moisture, with a significant difference in comparison to H13. Hybrid H5 from FAO 400 expressed a significant difference in grain moisture only in comparison to H7, while no substantial differences were seen in FAO 300 and 200. The comparison between FAO groups 200 and 300 showed a minimal difference in moisture content, with group 200 exhibiting a slightly higher moisture level compared to group 300. However, this difference was not statistically significant. Similarly, no significant difference in moisture content was observed between groups 200 and 400, with group 200 showing a slightly lower moisture level than group 400 (difference = −0.44%, *p* = 0.7773). In contrast, substantial differences in moisture content were observed between groups 200 and 500, as well as groups 200 and 600. Group 500 exhibited a significantly higher moisture level compared to group 200, while group 600 showed an even greater increase in moisture compared to group 200. Furthermore, comparisons between groups 300 and 400, groups 300 and 500, and groups 300 and 600 also revealed significant differences in moisture content. Group 400 showed a higher moisture level compared to group 300 (difference = −0.83%, *p* = 0.0347), while both groups 500 and 600 exhibited substantially higher moisture levels compared to group 300 (difference = −4.11%, *p* < 0.0001 and difference = −6.36%, *p* < 0.0001, respectively). Moreover, comparisons between groups 400 and 500, groups 400 and 600, and groups 500 and 600 demonstrated significant differences in moisture content. Group 500 exhibited a higher moisture level compared to group 400 (difference = −3.28%, *p* < 0.0001), while group 600 showed an even greater increase in moisture compared to group 400 (difference = −5.52%, *p* < 0.0001). Similarly, group 600 exhibited a higher moisture level compared to group 500 (difference = −2.24%, *p* < 0.0001).

**Figure 3:**
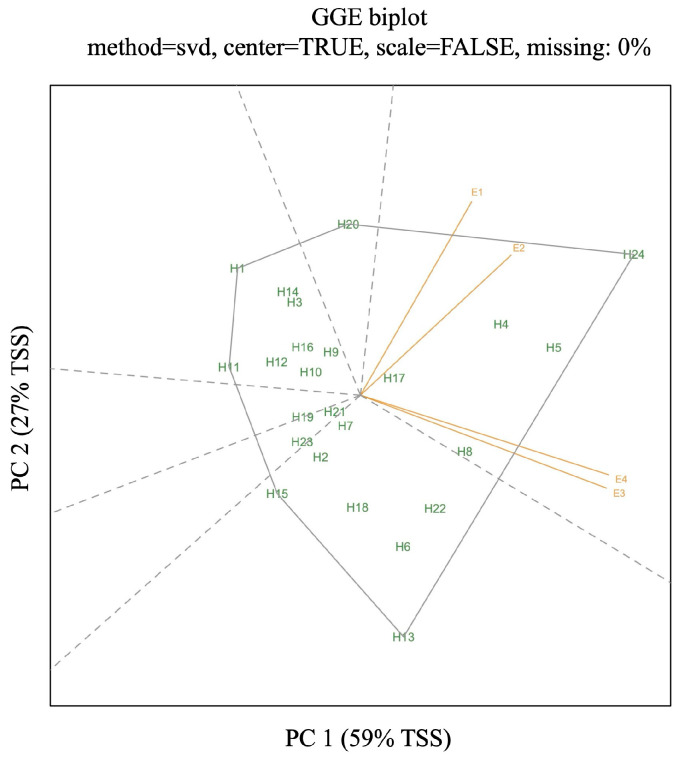
“Which–Won–Where” pattern analysis of hybrids. TSS = total sum of squares.

The Scree plot, which was used to determine the number of principal components and the contribution of each of them in Genotype + Genotype × Environment analysis (GGE), indicated that the first two components explained 86% of GGE variation (Figure 2a). The first and second principal components account for 59% and 27% of the variation, respectively. The dark-colored areas of the mosaic plot revealed that 55% of the total variation is illustrated by the differences between genotype means. The light-colored areas correspond to the variation due to G × E effects (43%) of the total variation. The panels of the mosaic plot correspond to principal component axes and indicate the first two IPCAs. This view of the mosaic plot indicated that the first IPCA had a very large contribution from the sum of squares of genotypes (SSG) and was considered a genotype axis, so that the distance between the points of a given genotype to this axis is highly correlated with the distances between the genotype means. The sum of squares of second IPCA had a much higher contribution to G × E effects than G effects and is labeled as a G × E interaction axis (Figure 2b). A heatmap was used for the investigation of Genotype + Genotype × Environment analysis, in which each column represented a different hybrid across environments and each row corresponded to a different environment for a given hybrid. Heatmap exhibited variations in the yield of hybrids across environments (Figure 2c). According to this heatmap plot, the most stable hybrid across all of the environments was H17. Also, H10 and H21 were stable across the environments, too. The highest yielding environment was E3, according to the heatmap. The “Which–Won–Where” biplot polygon was constructed of H1, H11, H13, H15, H20, and H24. It was divided into six sectors by rays perpendicular to the polygon sides (Figure 3). PC1 is on the *x*-axis and explains 59% of the variance in the data. PC2 is on the *y*-axis and accounts for 27% of the variance in the data. Environments in which the hybrids were tested are located in one sector. Genotype H24 is positioned farthest along the positive direction of all environment vectors, suggesting it consistently had the highest grain yield. The next highest yields are associated with genotypes H5 and H4, as determined by their relative positioning on the plot. Genotype H17 position near the origin indicates its stable performance across environments, with a more specific adaptation to the E2 as indicated by their closeness to each other. In contrast, genotypes H1 and H13 are located in the sectors opposite to environments E3 and E4, and E1 and E2, respectively, indicating they had the lowest yields in these environments. Genotype H8 proximity to E3 and E4 indicates that it is well adapted to these environments.

A regression analysis was conducted to investigate the relationship between planting density and yield by FAO group (Table 3). For the FAO 200 group, the slope coefficient, representing the average rate of change between two points on the parabola, was estimated at 0.188511 (*p* = 0.2999). Additionally, the quadratic coefficient, which captures the curvature of the relationship, was significant (*p* = 0.0375), estimated at −0.074503. Similarly, for the FAO 300 group, the average rate of change was estimated at 0.3469867 (*p* = 0.0275), and the quadratic coefficient was estimated at −0.093034 (*p* = 0.0027). For the FAO 400 group, the average rate of change was estimated at 0.6707859 (*p* = 0.0014), and the quadratic coefficient was estimated at −0.142305 (*p* = 0.0006). For the FAO 500 group, both the average rate of change and the quadratic coefficient were significant. Lastly, for the FAO 600 group, the average slope was estimated at 0.3188509 (*p* = 0.1667), and the quadratic coefficient was estimated at −0.084217 (*p* = 0.0635), meaning that FAO 600 grain yield was equal at different planting densities and did not depend on it. From average slopes, it seems that hybrids in the FAO 400 and FAO 500 groups had the highest response in yield with increasing planting densities.

**Figure 4:**
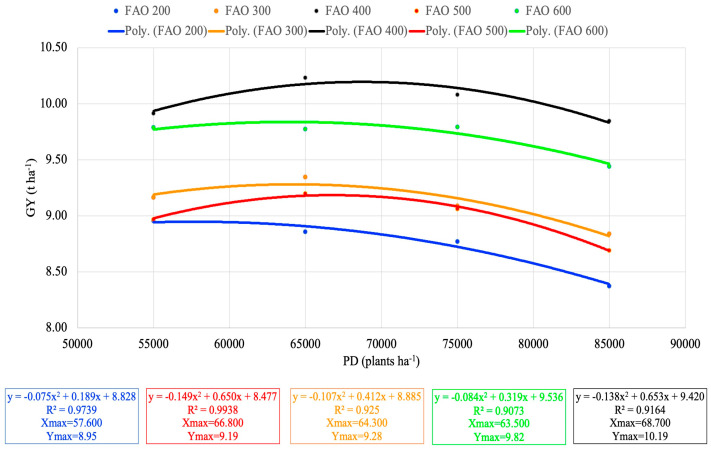
Regression model trendlines of grain yield depending on planting density, categorized by FAO groups. R^2^ = coefficient of determination; Xmax = estimated maximal planting density; Ymax = estimated maximal grain yield.

Solving the quadratic equations gives us insight into estimated maximum planting density (Xmax) necessary to achieve estimated maximum grain yield (Ymax) for each FAO group (Figure 4).

FAO 400 had the highest estimated yield (Ymax = 10.19 t ha^−1^), but to achieve this yield, the estimated planting density of 68,700 plants ha^−1^ was needed (Xmax = 68,700 plants ha^−1^). The second highest estimated yield was seen in the FAO 600 group, where an estimated planting density of 63,500 plants ha^−1^ was needed to achieve such a yield. Even though the estimated yield of FAO 300 was around 9% less than FAO 400, the estimated planting density was 64,400 plants ha^−1^ lower than FAO 400. According to Figure 4, the smallest estimated number of plants per ha to achieve the highest estimated yield was in FAO 200 (Xmax = 57,600 plants ha^−1^, Ymax = 8.95 t ha^−1^). The FAO 500 estimated maximal yield was 9.19 t ha^−1^ and was achieved with 66,800 plants ha^−1^.

## 3. Discussion

Plant density is one of the major factors that impact maize yield. The effect of density on yield and yield components has been studied from the early days of maize cultivation. The seeding density has changed during the last two decades with a tendency to increase the number of plants per unit area, which was contributed to by the appearance of new hybrids with better agronomic properties (e.g., higher strength of the lower internodes, upright position of the leaves, etc.), which, due to the changed architecture of the plant, could tolerate a denser stand [10,11,12]. In the 1930s, the commercial planting density of corn was 30,000 plant ha^−1^, and that number has risen to 80,000 plant ha^−1^ in the modern era. Maize hybrids of recent breeding cycles save water better, use mineral nutrients more rationally and efficiently, and tolerate denser sowing compared to previously created hybrids [13]. The physiological basis of yield improvement in maize hybrids grown at different plant densities has been well documented [14,15,16]. The physiological determinants responsible for genetic gain in maize grain yield have been associated with improved kernel number, enhanced postsilking biomass production, and biomass allocation to reproductive sinks [17,18,19]. A different number of plants per unit area leads to the change and interaction of a number of other factors (light intensity, degree of utilization of nutrients, efficiency of water absorption, radiation use efficiency in grain filling, etc.), whereby the “specific reaction” of the cultivated maize hybrid is determined by changing the quantity of yield. Hybrids with different leaf architecture and altered habitus can react differently to crop density, which is a consequence of variation in the number of leaves, plant height, leaf area per plant, and the vertical angle of the leaves in relation to the stem [20]. With increasing plant density, a reduced amount of solar radiation is intercepted by the lower strata leaves, and the utilization efficiency of radiation decreased, promoting an accelerated rate of leaf senescence [21,22,23]. High planting density usually causes increased plant height and ear height, resulting in root lodging, stem breaking, and yield loss [24]. The hybrids of the new generation respond favorably to higher crop density because they have a higher leaf area index (LAI) in the silking stage and the ability to absorb more photosynthetically active light, i.e., a higher light use efficiency [25]. Crop growth rate is directly related to the amount of RI (radiation intercepted) by the crop [26]. Dehdashti and Riahinia (2008) [27] studied the effect of different row spacing and density of maize on total day weight (TDW), leaf area index (LAI), net assimilation rate (NAR), and crop growth rate (CGR). Plot treatments were row spacing (60, 75, and 90 cm). Split plot treatments were within row spacing (12, 14, 16, and 18 cm). An increase of plant population (PP) from 10.5 to 13.9 plants m^−2^ increased LAI, TDW, and CGR but decreased NAR. Saberali et al. (2007) [28] investigated the effects of plant density and planting pattern on growth and physiological indices of maize (*Zea mays* L.). Plant density treatment was at two levels, i.e., recommended plant density (70,000 plant ha^−1^) and 1/5 times recommended plant density (105,000 plant ha^−1^). Planting pattern treatment was at two levels, i.e., one and two rows planting (planting on both ridge sides). The results showed that in high maize density, leaf area index, total dry weight, and crop growth rate increased more than low maize density in and throughout the growth season. Two-row planting patterns also increased leaf area index, total dry weight, and crop growth rate compared to one-row planting patterns, although it does not have the same effect as plant density. Valentinuz et al. [29] opined that the effect of increasing density on grain yield was greater under high incident radiation. The narrow row spacing could improve maize grain yield when maize is grown under less favorable growing seasons and suggested that the hybrids with different architectures respond similarly to plant density and row spacings. On average for all hybrids, the grain yield followed a second-degree polynomial model in response to the increasing planting density, with the highest value at PD2. To achieve maximum yield, the optimal planting density for maize hybrids in the FAO 200 group should be 57,600 plants ha^−1^, for the FAO 300 group 64,300 plants ha^−1^, for the FAO 400 group 68,700 plants ha^−1^, for the FAO 500 group 66,800 plants ha^−1^, and for the FAO 600 group 63,500 plants ha^−1^. The “Which–Won–Where” biplot showed that the hybrid H24 from FAO 600 group was the highest yielding in all of the environments. Hybrid H17 from the same FAO group was the most stable across all of the environments.

In the past decade, dryland maize production has become uncertain, especially in critical vegetation periods during summer months (July and August). Also, we are aware that we are increasingly faced with the occurrence of global climate changes, which pose new challenges to breeders on how to create hybrids with a strongly developed root system with the ability to retain accessible water in the soil. In stressful conditions caused by drought, maize plants sown in higher density significantly reduce grain yield, especially if the rainfall deficit is pronounced during the stage of flowering, fertilization, formation, and filling of grains [30,31]. On the other hand, some studies have shown that if the crop density increases over the optimal and/or recommended, the grain yield decreases due to the change of harvest index and increase of stalk length [32,33,34,35]. On more fertile soils with better physical–chemical and water–air properties, as well as in areas with a higher amount of precipitation during the growing season, denser sowing is possible, and vice versa, in drier regions, as well as on less fertile soils, sowing should be carried out with a lower seeding rate. Short-season maize hybrids are sown denser compared to late hybrids. Also, under irrigation conditions, sowing is denser. In mid-early and mid-late hybrids, the densities range from 57,000 to 68,000 plants ha^−1^, which are desirable, while in early hybrids, from 68,000 to 79,000 plants ha^−1^. Numerous studies have shown that if the crop density is increased over the optimal and/or recommended, the grain yield decreases due to changes of the harvest index and an increase of the stalk length [36,37]. Tollenaar and Lee [38] found that high plant density produced an increase in total dry matter production and a decrease in harvest index and that optimum plant density was a trade-off of both effects.

For many years, agricultural producers believed that the yield of maize depends on the yield per plant. However, new trends in modern maize production indicate that the increase in yield per unit area is largely dependent on the increase in plant density [39]. Determining the optimal seeding density is very risky, especially considering the fact that hybrids of recent breeding cycles form only one ear per plant [40]. Long-term studies in North America showed that in the period from 1930 to 1970, the average density of maize plants in the territory of the state of Minnesota was 19,000 plants ha^−1^ [41]. With the intensive application of mineral fertilizers, especially nitrogen, and modern production technology, the number of plants per unit area in the period from 1940 to 1990 was at the level of 5.6 plants m^−2^, while with the intensification and application of molecular genetics in the first decade of the 21st century, the number of plants rapidly increased to 7.1 plants m^−2^ [42]. Based on 30 years (1987–2016) of research and metadata obtained from the monitoring of 187,662 points on the territory of America, it was determined that when the yield increases, the density of plants increases in parallel with the increase in the number of cobs and the number of grains per plant. The agronomic optimum of plant density in the first analyzed study for the period from 1987 to 1991 was at the level of 75,000 plants ha^−1^, while in the period from 2012 to 2016, those values were within the limits of 93,000 plants ha^−1^. In the analyzed period, the yield increase was at the level of 149 kg ha^−1^ per year. The quadratic model in the mentioned study showed that with an increase in the agronomically optimal plant density, the yield also increased. By monitoring this phenomenon, it was observed that the rate of increase in yield versus density (according to the USDA) was similar in other major maize-producing states. Analyzes that include large databases of metadata indicate that the yield increase in modern hybrids is not only due to the plant density but also to the improvement of other factors, whereby the importance of the interaction of genotype (G) × environment (E) × management (M) is particularly emphasized [43,44].

## 4. Materials and Methods

### 4.1. Overview of Experimental Site Description

Field experiments were conducted at experimental field IFVCNS (two locations: Rimski Šančevi and Srbobran) to evaluate four plant densities (55,000; 65,000; 75,000; and 85,000 plants ha^−1^). The experimental sites “Rimski Šančevi” and “Srbobran” are located in the typical chernozem zone of the southern part of the Pannonian Basin. The size of the experimental plot was 30 m^2^, with 10 m in length and 4 rows with 0.75 m between rows wide. The experiment was in split-plot design with 3 replications, in which whole plots were PD, and subplots were hybrids. The details of the locations and treatments are shown in Table 4.

### 4.2. Agronomic Management

Conventional tillage, including moldboard plowing, disc harrowing, and field cultivating, was performed, and harvest residues were plowed under. The N fertilizer used in the experiment was urea (46.4% N). Fertilizer KAN-27% was applied at rates of 100 kg ha^−1^ in two splits at 3 leaves and 6–8 leaves. The P and K fertilizers were superphosphate and potassium chloride with dosages of 40.0 and 55 kg ha^−1^. Half of N and all P and K fertilizer were applied as base fertilizer, and half of N fertilizer was applied in the V10 stage. In both localities, the preceding crop was winter wheat. Grain maize yield was calculated as an average of three replicates and adjusted to 14% moisture content for maize.

### 4.3. Climatic Data

**Figure 5:**
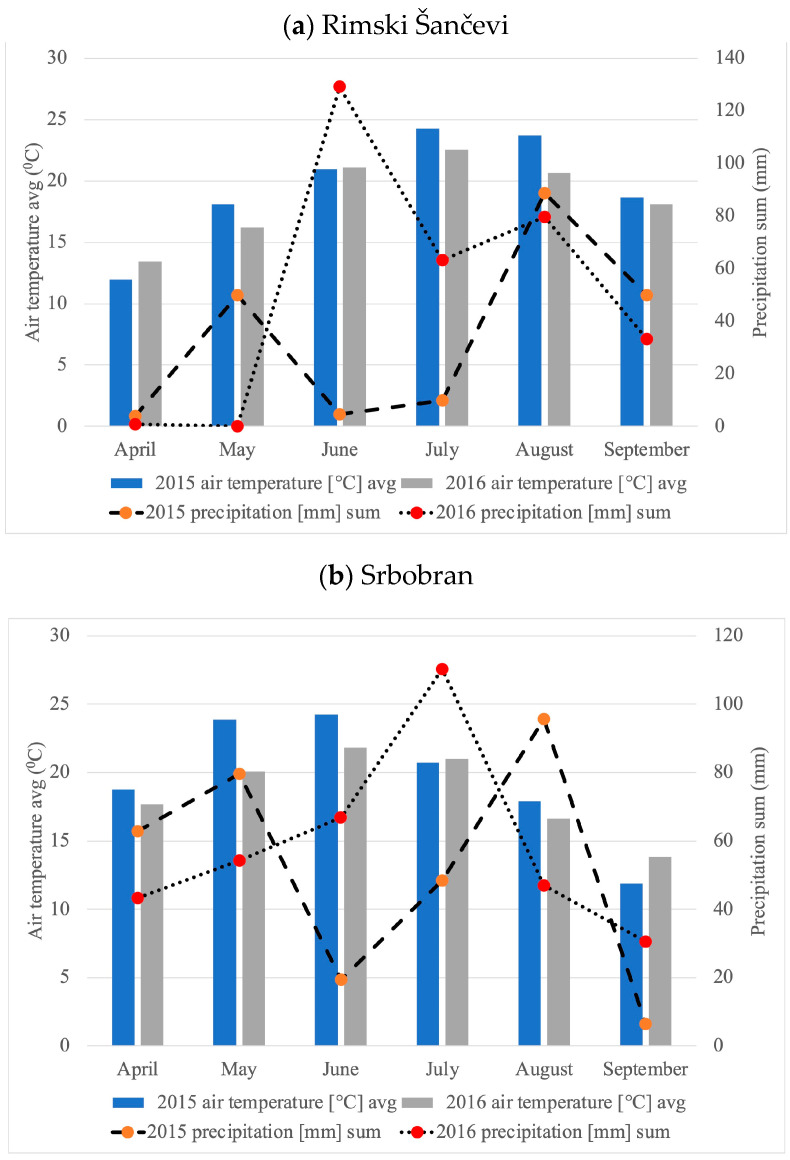
Weather conditions in Rimski Šančevi (**a**) and Srbobran (**b**) in 2015 and 2016.

Weather conditions in the South Pannonian Basin are variable and influence maize yield. The long-term average annual precipitation and average temperatures at the study site are 416 mm, 17.2 °C (Rimski Šančevi) and 451 mm, 16.2 °C (Srbobran), respectively. In the observed years, it can be noticed that the annual amount of precipitation and precipitation in the vegetation period has increased. However, compared to multi-year values, the amount of precipitation was significantly reduced in August (2013), as well as during June (2014 and 2015) and July (2013 and 2015). Weather conditions during the experiment period are presented in Figure 5.

### 4.4. Statistical Analysis

For testing the significance of main factors and their interaction on grain yield and grain moisture content at harvest time during the period from 2015 to 2016, a linear mixed model (LMM) was employed. The fixed effects chosen were hybrids, planting density, and hybrids × planting density, while the random effects selected were repetitions nested within locations and year (repetitions [locations, year]) (Equation (1)).

(1) GYijkl=μ+Hi+PDj+(H×PD)ij+Rk(LlYm)+ϵijkl

where GY*ijkl* is the grain yield for the *i*-th hybrid (H), *j*-th planting density (PD), *k*-th repetition, *l*-th location, and *m*-th year; *μ* is the overall mean yield; H*i* is the fixed effect of the *i*-th hybrid; PD*j* is the fixed effect of the *j*-th planting density; hybrid × PD*ij* is the interaction effect between the *i*-th hybrid and *j*-th planting density; R*k*(L*l*Y*m*) represents the random effect of the *k*-th repetition nested within the *l*-th location and the *m*-th year; and ϵ*ijkl* is the residual error term. Tukey’s honestly significant difference (HSD) test for multiple comparisons was used for comparing means of groups among treatments. Both the LMM and multiple comparison tests were conducted at a significance level of 5%. Regression analysis was performed to ascertain the relationship between grain yield and planting density. Genotype + Genotype × Environment methodology was used for visual analysis of the contribution of both genotype (G) and genotype–environment interaction (GE) factors for genotypic evaluation for grain yield response of 24 maize hybrids at 4 different environments. Environments were depicted as E1 (2015 Rimski Šančevi), E2 (2015 Srbobran), E3 (2016 Rimski Šančevi), and E4 (2016 Srbobran). Statistical data processing was carried out using the JMP ^®^ Pro 14.0.0 statistical software and R 4.3.2 statistical software. The GGE biplot was generated using the gge package in R [45]. The model was specified with Yield ~ Genotype × Environment. The scaling was not applied to the data, while convex hull was added to the plot, which helps in identifying the extremes of the genotypes and environments. In addition to the biplot, partitioning of the sums-of-squares simultaneously along the principal component axes and along the G and GE axes was performed [46]. A heatmap was also generated to visualize the interaction effects across different genotypes and environments.

## 5. Conclusions

Grain yield varied with plant density and maize hybrids. The planting density to achieve a high yield of maize was relatively broad, and the density for maximum yield in different maize planting environments was related to climate, soil, and yield potential. Twenty-four maize hybrids belonging to five maturity groups, all with four different plant densities, were tested in two habitats. On average for all hybrids, the grain yield followed a second-degree polynomial model in response to the increasing planting density, with the highest value at 65,000 plant ha^−1^. The FAO 400 maturity group gave the most outstanding grain yield. To achieve maximum yield, the optimal planting density for corn hybrids of the FAO 200 group should be 57,600 plants ha^−1^, for the FAO 300 group 64,300 plants ha^−1^, for the FAO 400 group 68,700 plants ha^−1^, for the FAO 500 group 66,800 plants ha^−1^, and for the FAO 600 group 63,500 plants ha^−1^. The “Which–Won–Where” biplot showed that the hybrid H24 from FAO 600 group was the highest yielding in all of the environments. Hybrid H17 from the same FAO group was the most stable across all of the environments. Selected hybrids may further be studied for planting density and nutritional requirements for getting maximum yield. Future research should take into account that the connection between plant density and yield is a fundamental relationship and that in future analysis of the influence of any factor on yield, this relationship must not be neglected. Modern and sustainable maize production should go in the direction of increasing the sowing density, whereby the number of ears per plant, the number of grains per ear, and the 1000-grain weight will play an important role in determining the potential of cultivated hybrids. The development of earlier hybrids, with shorter plant height, lower leaf number, upright leaves, smaller tassels, and more synchronized floral development, can improve maize’s ability to withstand high plant densities without presenting a higher percentage of barren plants.

## Figures and Tables

**Table 1:** Effect of hybrids, planting density, and their interaction on the variability of grain yield (a) and moisture (b) from the 2015 and 2016 seasons. **, significant (*p* < 0.01), ns, non-significant.

(**a**)					
Source	Nparm	DFNum	DFDen	F Ratio	Prob > F
Hybrid (H)	23	23	1043.0	15.750	<0.0001 **
Planting density (PD)	3	3	1043.0	5.001	0.0019 **
Hybrid (H) × Planting density (PD)	69	69	1043.0	0.707	0.9657 ^ns^
(**b**)					
Source	Nparm	DFNum	DFDen	F Ratio	Prob > F
Hybrid (H)	23	23	1043.0	60.643688	<0.0001 **
Planting density (PD)	3	3	1043.0	0.8314738	0.4766 ^ns^
Hybrid (H) × Planting density (PD)	69	69	1043.0	0.4710766	0.9999 ^ns^

Nparm―the number of parameters that are associated with the effect; DFNum—the numerator degrees of freedom for the effect test; DFDen—the degrees of freedom for error.

**Table 2:** Grain yield (GY) differences depending on planting density, together with regression model coefficients. **, significant (*p* < 0.01).

Planting Density (PD)	GY (t ha^−1^)	Std Error	Lower 95%	Upper 95%
PD1 ab	9.50	0.54	8.31	10.68
PD2 a	9.65	0.54	8.46	10.83
PD3 a	9.55	0.54	8.37	10.74
PD4 b	9.23	0.54012569	8.05	10.42
**Parameter**	**GY (t ha^−1^)**	**Prob > Chi Square**	**Lower 95%**	**Upper 95%**
Intercept	9.11	<0.0001 **	9.08	9.14
Slope	0.50	<0.0001 **	0.48	0.53
Quadratic	−0.12	<0.0001 **	−0.12	−0.11

**Table 3:** Regression model coefficients of grain yield depending on planting density, categorized by FAO groups. **, significant (*p* < 0.01), *, significant (*p* < 0.05), ns: non-significant.

FAO Group	Parameter	Estimate	Prob > Chi Square	Lower 95%	Upper 95%
	Intercept	8.8280847	<0.0001 **	8.4373621	9.2188072
FAO 200	Slope	0.188511	0.2999 ^ns^	−0.167938	0.5449601
	Quadratic	−0.074503	0.0375 *	−0.144679	−0.004327
	Intercept	8.9275037	<0.0001 **	8.5894006	9.2656069
FAO 300	Slope	0.3469867	0.0275 *	0.0385414	0.6554321
	Quadratic	−0.093034	0.0027 **	−0.153759	−0.032309
	Intercept	9.4059601	<0.0001 **	8.955744	9.8561762
FAO 400	Slope	0.6707859	0.0014 **	0.2600619	1.0815098
	Quadratic	−0.142305	0.0006 **	−0.223167	−0.061444
	Intercept	8.4767842	<0.0001 **	8.317291	8.6362773
FAO 500	Slope	0.6502111	<0.0001 **	0.5047084	0.7957137
	Quadratic	−0.149373	<0.0001 **	−0.178019	−0.120728
	Intercept	9.5357623	<0.0001**	9.0404337	10.031091
FAO 600	Slope	0.3188509	0.1667 ^ns^	−0.133028	0.7707301
	Quadratic	−0.084217	0.0635 ^ns^	−0.173181	0.0047464

**Table 4:** Experimental sites, sowing dates, and maize (*Zea mays* L.) plant densities in the experiments in different locations.

Experimental Sites	Geographic Coordinates	Sowing Date	Hybrid	Plant Density (Plants ha^−1^)	Soil Type
Rimski Šančevi	45°19′44.3″ N 19°49′40.7″ E	14 April	24 hybrids (H1–H24) FAO 200 (H1) FAO 300 (H2–H3) FAO 400 (H4–H9) FAO 500 (H10–H16) FAO 600 (H17–H24)	PD1: 55,000 PD2: 65,000 PD3: 75,000 PD4: 85,000	Chernozem
Srbobran	45°31′59″ N 19°47′34″ E	21 April	24 hybrids (H1–H24) FAO 200 (H1) FAO 300 (H2–H3) FAO 400 (H4–H9) FAO 500 (H10–H16) FAO 600 (H17–H24)	PD1: 55,000 PD2: 65,000 PD3: 75,000 PD4: 85,000	Chernozem

## Data Availability

Data will be made available on demand.
